# The association of inflammatory biomarkers with clinical outcomes in diabetic retinopathy participants: data from NHANES 2009–2018

**DOI:** 10.1186/s13098-024-01419-4

**Published:** 2024-07-29

**Authors:** Yueqiao Si, Qingwei Chen, XiaoJing Xiong, Minming Zheng

**Affiliations:** 1 Department of General Practice, The Second Affiliated Hospital of Chongqing Medical University, Chongqing, 400010 China; 2 Department of Ophthalmology, The Second Affiliated Hospital of Chongqing Medical University, Chongqing, 400010 China

**Keywords:** Diabetic retinopathy, Neutrophil to lymphocyte ratio, Monocyte to lymphocyte ratio, System inflammation response index, Mortality

## Abstract

**Objective:**

The aim of this study was to assess the association of neutrophil lymphocyte ratio (NLR), monocyte to lymphocyte ratio (MLR), and system inflammation response index (SIRI) with the all-cause mortality and diabetes-cardiovascular mortality in participants with diabetic retinopathy (DR).

**Methods:**

A total of 572 participants with DR from NHANES were included, and divided into survival group (*n* = 440) and all-cause death group (*n* = 132). NLR = neutrophil count/lymphocyte count, MLR = monocyte count/lymphocyte count, SIRI = (neutrophil count × monocyte count)/lymphocyte count. We utilized the NHANES Public-Use Linked Mortality File through April 26, 2022, to determine mortality status. Diabetes-cardiovascular death was defined as death resulting from heart disease, cerebrovascular disease, or diabetes mellitus. The Spearson Correlation Analysis, Kaplan-Meier curves, Cox proportional hazards regression models, Restricted cubic spline plots and Decision Curve Analysis were used.

**Results:**

The all-cause mortality and diabetes-cardiovascular mortality were significantly higher in NLR ≥ 1.516, MLR ≥ 0.309, SIRI ≥ 0.756, and NLR + MLR + SIRI subgroups than NLR < 1.516, MLR < 0.309, SIRI < 0.756 subgroups, and other participants except NLR + MLR + SIRI (all *P* < 0.05). The HR of NLR, MLR, SIRI, NLR + MLR + SIRI for all-cause mortality were 1.979(1.13–3.468), 1.850(1.279–2.676), 1.821(1.096–3.025), 1.871(1.296–2.703), respectively. The hazard ratio of NLR, MLR, SIRI, NLR + MLR + SIRI for diabetes-cardiovascular mortality were 2.602(1.028–6.591), 2.673(1.483–4.818), 2.001(0.898–4.459), 2.554(1.426–4.575), respectively. In the restricted cubic spline plots, the relationship between NLR, MLR, SIRI and HR of all-cause mortality and diabetes-cardiovascular mortality was overall as “J” shaped. In both age < 60 and age > 60 years participants, the all-cause mortality and diabetes-cardiovascular mortality were significantly higher in NLR ≥ 1.516, MLR ≥ 0.309, SIRI ≥ 0.756, and NLR + MLR + SIRI subgroups than NLR < 1.516, MLR < 0.309, SIRI < 0.756 subgroups, and other participants except NLR + MLR + SIRI (all *P* < 0.05).

**Conclusion:**

NLR, MLR, and SIRI may be three independent prognostic predictors for all-cause mortality and diabetes-cardiovascular mortality among individuals with DR. In practical clinical applications, combining NLR, MLR, and SIRI may enhance the prediction of all-cause mortality and diabetes-cardiovascular mortality in DR.

**Supplementary Information:**

The online version contains supplementary material available at 10.1186/s13098-024-01419-4.

## Introduction

Diabetic retinopathy (DR) is a prominent microvascular complication that threatens vision in approximately 30–40% of diabetic patients [1–3]. The global prevalence and burden of DR have escalated due to the growing prevalence of diabetes, lifestyle changes, and an aging population [4]. DR, a severe late-stage complication of diabetic patients, is associated with increased severity and higher mortality. With improvements in the quality of life and an aging population, enhancing patient quality and lifespan extension have become key areas of current research.

The progression and development of DR can vary and do not always correlate with blood sugar control [5]. Chronic inflammation plays a pivotal role in the pathological mechanism of DR, affecting both type 1 and type 2 diabetes. DR occurs within the context of chronic systemic inflammation, where circulating inflammatory mediators alter retinal functions [6]. Systemic inflammation and oxidative stress are significant challenges in the pathogenesis mechanism of DR [7, 8]. The inflammatory response, a non-specific response to injury or stress, is associated with insulin resistance and the destruction of pancreatic beta cells [9]. In diabetes, chronic inflammation can activate inflammatory cells, leading to the secretion of inflammatory substances that contribute to capillary dysfunction and exacerbation of DR [10–12]. Previous studies have shown that anti-diabetic drugs, especially metformin, may lower chronic inflammation and reduce the risk of inflammatory diseases in T2DM patients [13, 14].

Neutrophil lymphocyte ratio (NLR), monocyte-to-lymphocyte ratio (MLR), and the systemic inflammation response index (SIRI) serve as complex, cost-effective, and easily calculable biomarkers of inflammation. These biomarkers are widely used as prognostic biomarkers for various diseases, including diabetes, pneumonia, cardiovascular disease, and malignancies [15–17]. However, few studies have explored the relationship between NLR, MLR, SIRI, and diabetes and its complications. Currently, studies have yet to investigate the potential of three key inflammatory biomarkers as predictive indicators for DR. Understanding the correlation between these biomarkers and DR could facilitate the development of early intervention strategies, thereby enhancing clinical outcomes and improving patient prognosis. The National Health and Nutrition Examination Survey (NHANES) is the most in-depth survey designed to evaluate the health and nutritional status of adults and children in the United States, including studies on diabetes, retinopathy, and other factors critical to health. This study aimed to assess the association of NLR, MLR, and SIRI with all-cause mortality and diabetes-cardiovascular mortality in participants with DR baes on the NHANES database.

## Methods

### Study population

**Fig. 1:**
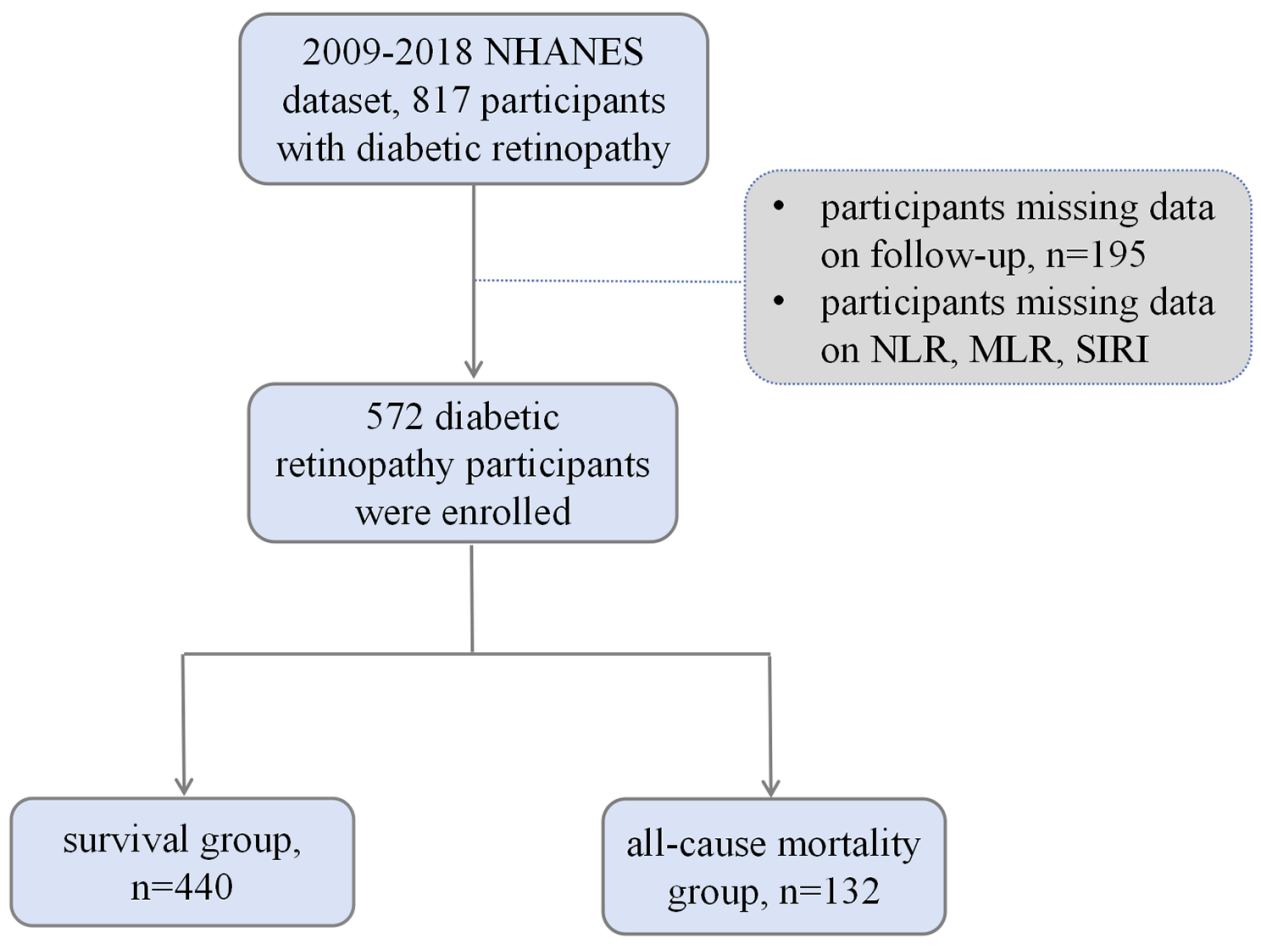
Flow charts for participants in this study

We utilized data from NHANES for 10 years (2009 to 2018), of them, 623 participants with DR. Participants with missing data on follow-up data (*n* = 195) and NLR, MLR, SIRI (*n* = 50) were excluded. Finally, a total of 572 DR participants were enrolled in this study, and they were divided into survival group (*n* = 440) and all-cause death group (*n* = 132) (Fig. 1). Details about NHANES are provided through the Centers for Disease Control and Prevention (https://www.cdc.gov/nchs/nhanes/index.htm). NHANES has been approved by the NCHS Ethics Review Board.

NHANES is a reliable and high-quality database, which including medical information, physiological measurements, and laboratory tests supervised by trained medical personnel [18]. Then, we calculated the value of NLR, MLR, and SIRI according to the following equations: NLR = neutrophil count/lymphocyte count, MLR = monocyte count/lymphocyte count, SIRI = (neutrophil count × monocyte count)/lymphocyte count [16, 19, 20]. DR was confirmed using a dichotomous, self-reported item, indicating that a doctor had informed the respondent that diabetes had affected their eyes.

### Outcome ascertainment

We utilized the NHANES Public-Use Linked Mortality File through April 26, 2022, linked by the National Death Index with a probabilistic matching algorithm to determine mortality status [21]. The National Death Index is an NCHS centralized database encompassing all deaths in the US. All-cause of death of the NCHS classified mortality from heart diseases (codes 054–068), malignant neoplasms (codes 019–043), chronic lower respiratory diseases (codes 082–086), unintentional injuries (codes 112–123), cerebrovascular diseases (codes 070), Alzheimer’s disease (codes 052), diabetes mellitus (codes 046), influenza and pneumonia (codes 076–078), nephritis, nephrotic syndrome and nephrosis (codes 097–101), all other causes (residual). In this study, diabetes-cardiovascular death was defined as death resulting from heart disease (*n* = 49), cerebrovascular disease (*n* = 4), or diabetes mellitus (*n* = 4). Participants follow up until death or on April 26, 2022, for survivors.

### Statistical analysis

Continuous variables were presented as quartile M (QR), with categorical variables expressed as percentages. NHANES recommended weights were applied to account for the planned oversampling of specific groups. Spearson correlation analysis examined the relationships between clinical variables (age, systolic blood pressure, diastolic blood pressure, urinary creatinine to albumin ratio, total cholesterol, low density lipoprotein cholesterol, triglyceride, high density lipoprotein cholesterol, white blood cell, red blood cell, hemoglobin, red cell distribution width, platelet count, glycosylated hemoglobin) and NLR, MLR, and SIRI. Receiver operating characteristic (ROC) curve analyses were conducted for all-cause death to determine the cut-off values for NLR, MLR, and SIRI, allowing for a comparison of their diagnostic efficiency. Instances, where NLR + MLR + SIRI all exceeded their cut-off values, were donated as NLR, MLR, and SIRI. Kaplan-Meier curves were plotted for all-cause mortality and diabetes-cardiovascular mortality in subgroups with NLR, MLR, SIRI ≥ cut-off value, and < cut-off value. Additional Kaplan-Meier curves were generated for participants with NLR + MLR + SIRI and others. The associations between NLR, MLR, SIRI, NLR + MLR + SIRI, and all-cause mortality diabetes-cardiovascular mortality were investigated using Cox proportional hazards regression models with covariates. Kaplan-Meier curves of all-cause mortality in ages < 60 years and age ≥ 60 were also plotted for NLR, MLR, SIRI, and NLR + MLR + SIRI.

Restricted cubic spline plots were employed to explore the association between NLR, MLR, SIRI, and all-cause mortality in patients with DR. The decision curve analysis curves assessed the efficacy of NLR, MLR, SIRI, and NLR + MLR + SIRI for clinically predicting all-cause mortality in patients with DR. Both restricted cubic spline plots and decision curve analysis curves were created using the R version 4.2.3.

## Results

A total of 572 participants were included (unweighted *n* = 132 all-cause mortality for DR [weighted 26.6%]). Characteristics of the study participants between survival and all-cause death groups are presented in Table 1. Statistically significant differences in gender, age distribution, body mass index, hypertension, cardiovascular disease, hypercholesterolemia, renal failure, NLR, MLR, and SIRI (all *P* < 0.001). Clinical examination and laboratory findings between the two groups are shown in Table 2. Levels of systolic blood pressure, urinary creatinine to albumin ratio, red cell distribution width, NLR, MLR, and SIRI were higher in the all-cause mortality group than in the survival group (all *P* < 0.05). Meanwhile, diastolic blood pressure, total cholesterol, low-density lipoprotein cholesterol, lymphocyte, red blood cell, hemoglobin, and platelet count were higher in the survival group than in the all-cause death group (all *P* < 0.05).

**Table 1:** Characteristics of participants between survival and all-cause death groups

	Survival Unweighted sample size (percentage**)	All-cause mortality Unweighted sample size (percentage**)	P*
Total, million (%)	0.128 (73.4)	0.046 (26.6)	
Gender			< 0.001
Male	208(54.6)	55(63.9)	
Female	232(45.4)	77(36.1)	
Age			< 0.001
< 45 years	197(49.1)	19(10.3)	
45–60 years	221(46.6)	80(63.4)	
≥ 60 years	22(4.3)	33(26.2)	
Body mass index			< 0.001
< 30 kg/m2	198(44.1)	60(58.1)	
≥ 30 kg/m2	242(55.9)	72(41.9)	
Combined disease			
Hypertension	313(70.9)	109(78.8)	< 0.001
Cardiovascular disease	143(35.9)	50(34.2)	< 0.001
Hypercholesterolemia	273(65.7)	90(70.9)	< 0.001
Renal failure	59(17.0)	27(23.1)	< 0.001
NLR			< 0.001
< 1.516	105(75.5)	14(92.5)	
≥ 1.516	335(24.5)	118(7.5)	
MLR			< 0.001
< 0.309	289(38.2)	54(57.5)	
≥ 0.309	151(61.8)	78(42.5)	
SIRI			< 0.001
< 0.756	112(24.0)	18(12.1)	
≥ 0.756	328(76.0)	114(87.9)	

*P was based on design-based χ2 test; **weighted percentage

NLR, neutrophil lymphocyte ratio; MLR, monocyte to lymphocyte ratio; SIRI, system inflammation response index

**Table 2:** Clinicall examination and laboratory

	Survival (*n* = 440)	All-cause mortality (*n* = 132)	Z	*P*
Systolic blood pressure(mmHg)	132(120–146)	138(122–151)	-2.762	0.006
Diastolic blood pressure(mmHg)	70(62–78)	63(53–72)	-3.304	0.001
Urinary creatinine to albumin ratio (mg/g)	22.8(8.5-105.4)	68.7(14.9-242.7)	-5.223	< 0.001
Total cholesterol (mg/dL)	173(147–207)	166(142–192)	-2.985	0.003
Low density lipoprotein cholesterol (mg/dL)	98(75–125)	91(65–114)	-2.263	0.024
Triglyceride(mg/dL)	115(78–177)	120(87–151)	-0.804	0.421
High density lipoprotein cholesterol (mg/dL)	47(40–56)	47(41–58)	-0.461	0.645
White blood cell (109/L)	6.8(5.8–8.1)	7.3(5.9–8.9)	-1.285	0.199
Lymphocyte(%)	1.8(1.6–2.3)	1.7(1.1–2.2)	-5.074	< 0.001
Monocyte(%)	0.5(0.4–0.7)	0.6(0.5–0.7)	-0.495	0.621
Neutrophils(%)	4(3.3–5.2)	4.7(3.8-6.0)	-0.493	0.622
Red blood cell(1012/L)	4.57(4.16–5.02)	4.26(3.78–4.70)	-5.359	< 0.001
Hemoglobin (g/dL)	13.6(12.7–15.0)	13.0(11.5–13.8)	-5.128	< 0.001
Red cell distribution width (%)	13.4(12.6–14.2)	13.5(12.7–14.3)	-3.166	0.002
Platelet count (109/L)	226(194–268)	211(152–266)	-3.656	< 0.001
Glycosylated hemoglobin(%)	7.4(6.5–8.85)	7.5(6.3–8.7)	-0.827	0.408
NLR	2.095(1.666-3.000)	2.363(1.829–4.163)	-4.555	< 0.001
MLR	0.2759(0.192–0.372)	0.317(0.244–0.429)	-5.020	< 0.001
SIRI	1.067(0.723–1.713)	1.418(0.849–2.187)	-3.719	< 0.001
Insulin (%)	133(47.5)	64(57.7)	1.505	0.070
Antidiabetc drugs (%)	188(53.9)	63(51.2)	0.899	0.613
Antihypertensive drugs (%)	305(72.8)	108(85.0)	2.125	0.005

Spearson correlation analysis revealed that NLR, MLR, and SIRI were all positively or negatively correlated with age (0.129, 0.220, and 0.101, respectively), diastolic blood pressure (–0.169, − 0.135, 0.137, respectively), total cholesterol (–0.164, − 0.249, 0.194, respectively), and red cell distribution width (0.129, − 0.157, 0.195, respectively) (all *P* < 0.05). Additional variables related to NLR, MLR, and SIRI are detailed in Table 3.

**Table 3:** The Spearson correlation analysis of NLR, MLR, and SIRI

	NLR		MLR		SIRI	
	*r*	*P*	*r*	*P*	*r*	*P*
Age	0.129	0.002	0.220	< 0.001	0.101	0.016
Systolic blood pressure	-0.090	0.036	-0.041	0.339	-0.097	0.023
Diastolic blood pressure	-0.169	< 0.001	-0.135	0.002	-0.137	0.001
Urinary creatinine to albumin ratio	0.112	0.009	0.068	0.113	0.092	0.033
Total cholesterol	-0.164	< 0.001	-0.249	< 0.001	-0.194	< 0.001
Low density lipoprotein cholesterol	-0.112	0.088	-0.178	0.007	-0.152	0.021
Triglyceride	-0.044	0.498	-0.174	0.007	-0.073	0.259
High density lipoprotein cholesterol	-0.063	0.135	-0.025	0.555	-0.112	0.008
White blood cell	0.199	< 0.001	-0.048	0.255	0.461	< 0.001
Red blood cell	-0.145	0.001	-0.143	0.001	-0.063	0.133
Hemoglobin	-0.113	0.007	-0.059	0.160	-0.043	0.305
Red cell distribution width	0.129	0.002	0.157	< 0.001	0.195	< 0.001
Platelet count	-0.095	0.022	-0.212	< 0.001	0.011	0.795
Glycosylated hemoglobin	-0.069	0.099	-0.170	< 0.001	-0.066	0.117

**Fig. 2:**
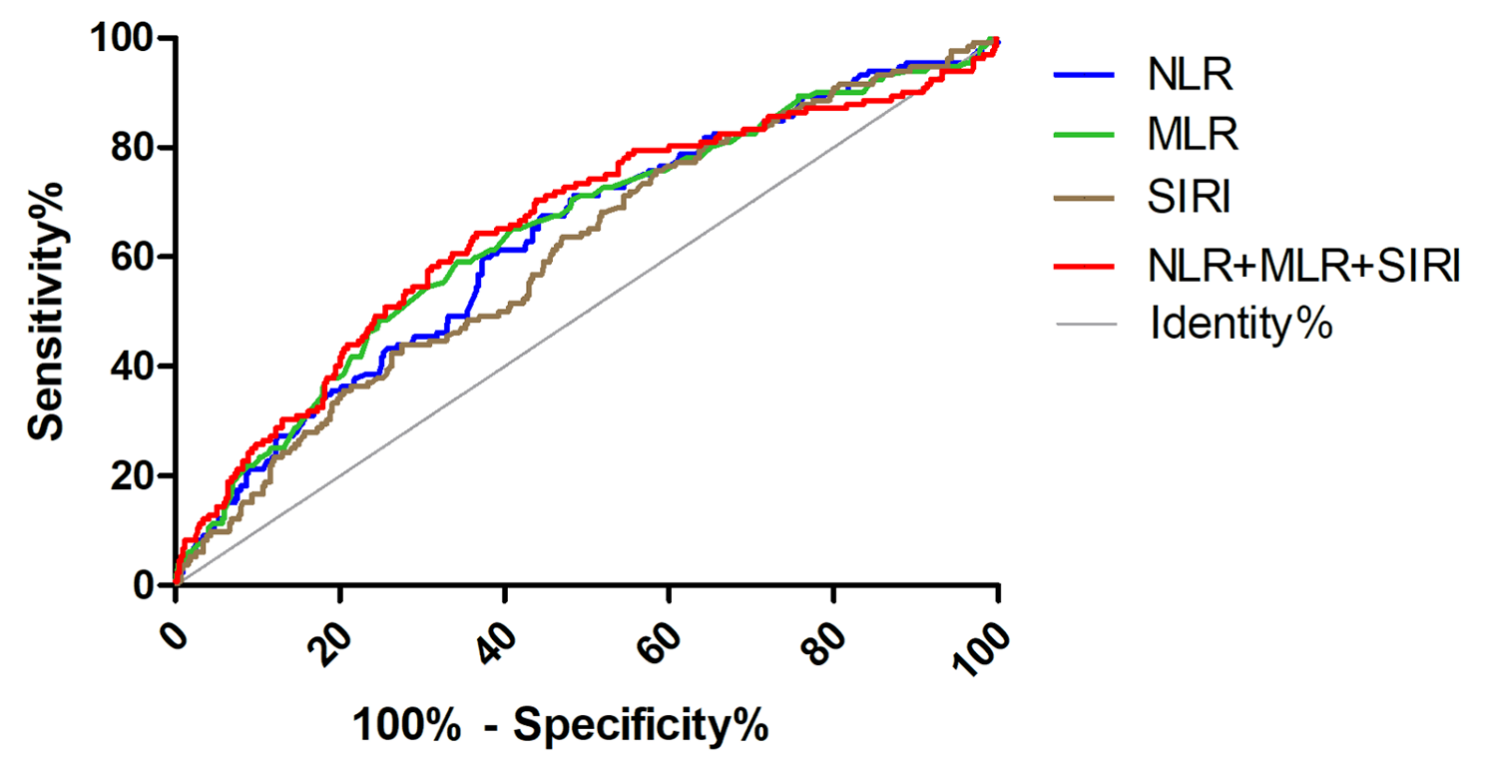
The ROC curve analyses for NLR, MLR, SIRI, and NLR + MLR + SIRI of all-cause death

In ROC curve analyses, the AUC of NLR, MLR, SIRI, and NLR + MLR + SIRI were 0.613 (0.577–0.685), 0.644 (0.589–0.699), 0.607 (0.552–0.661), and 0.653 (0.597–0.709), respectively. The cut-off values for NLR, MLR, and SIRI were 1.516, 0.309, and 0.756, respectively (Fig. 2; Table 4). The sensitive, specificity, positive, and negative predictive values are shown in Table 4. NLR and SIRI exhibited higher sensitivity, while MLR and NLR + MLR + SIRI had higher specificity. The positive predictive value of these metrics was higher than the negative predictive value. Decision curve analysis compared the clinical effectiveness of NLR, MLR, SIRI, and NLR + MLR + SIRI. MLR and NLR + MLR + SIRI were slightly better than NLR and SIRI for all-cause mortality (Supplementary Fig. 1).

**Table 4:** The ROC curve analyses for NLR, MLR, and SIRI of all-cause mortality

Variables	AUC	*P*	Sensibility(%)	Specificity (%)	Positive Predictive Value (%)	Negative Predictive Value (%)	cut-off vaule
NLR	0.613(0.577–0.685)	< 0.001	89.4	23.9	26.0	11.8	1.516
MLR	0.644(0.589–0.699)	< 0.001	59.1	65.7	34.1	15.7	0.309
SIRI	0.607(0.552–0.661)	< 0.001	89.4	20.2	25.8	13.8	0.756
NLR + MLR + SIRI	0.653(0.597–0.709)	< 0.001	58.3	66.4	34.2	15.9	-

**Fig. 3:**
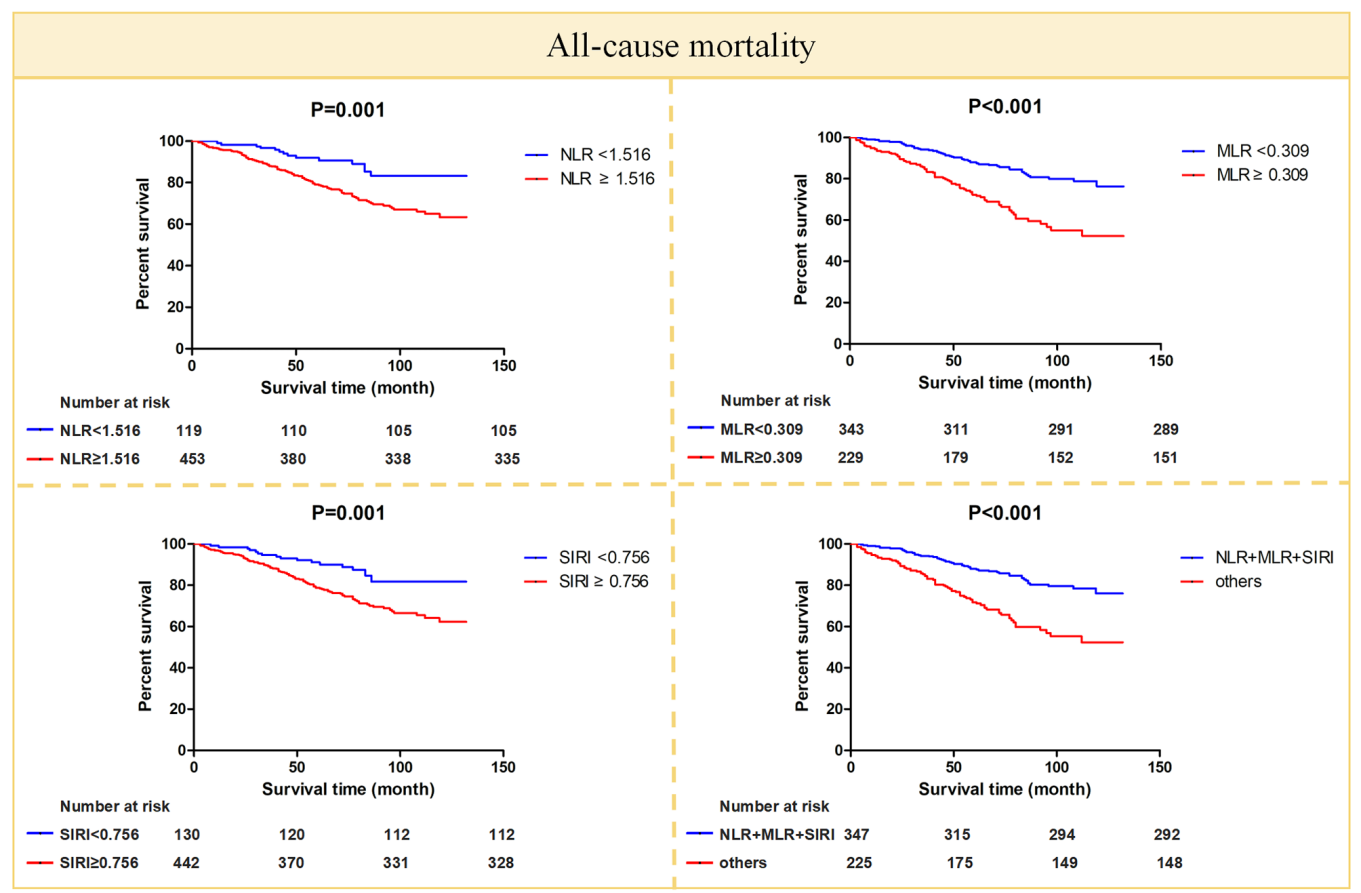
The Kaplan-Meier curves for all-cause mortality for NLR, MLR, SIRI and NLR + MLR + SIRI, respectively

**Fig. 4:**
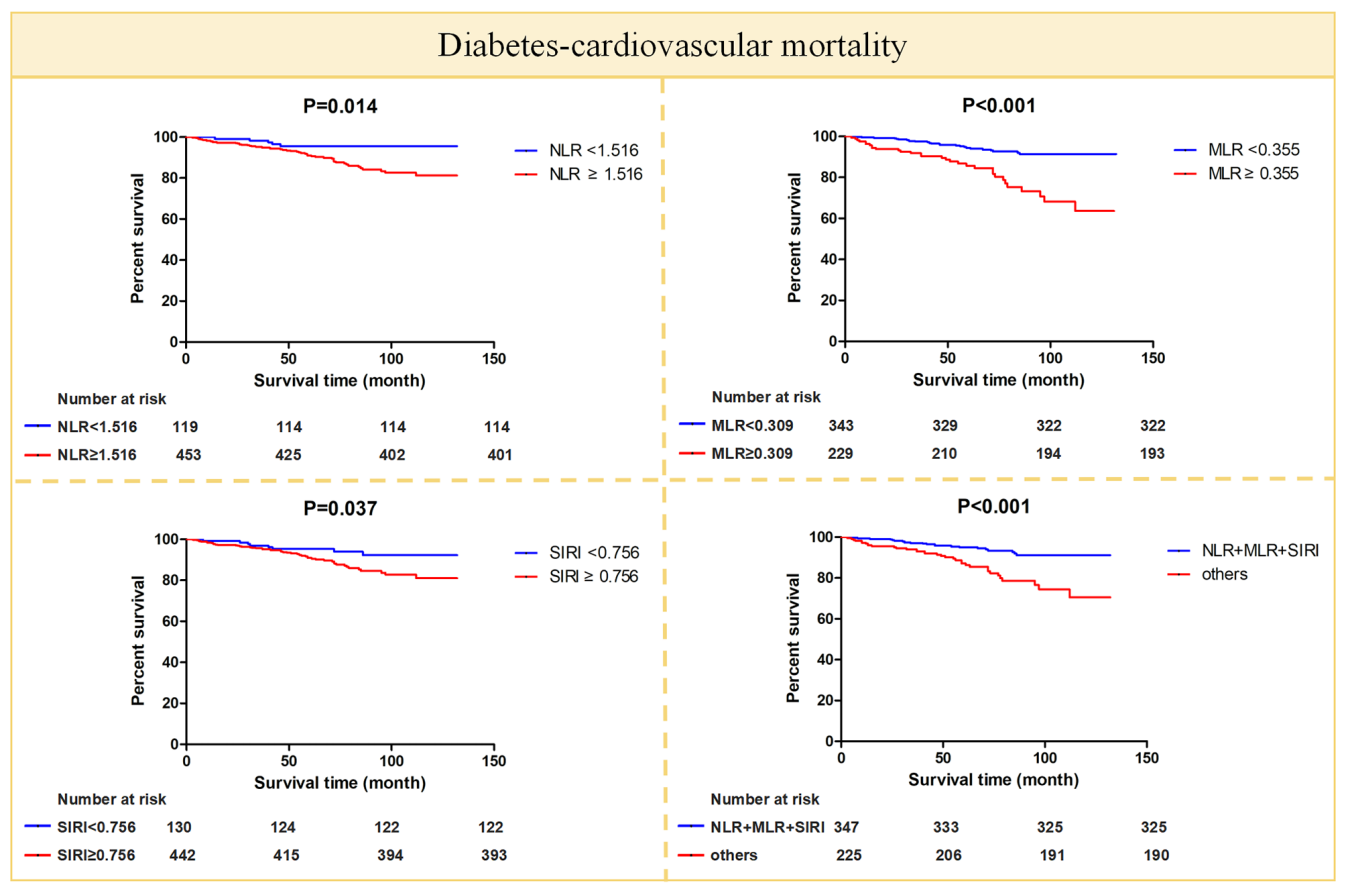
The Kaplan-Meier curves for diabetes-cardiovascular mortality for NLR, MLR, SIRI and NLR + MLR + SIRI, respectively

To explore the relationship of NLR, MLR, SIRI, and NLR + MLR + SIRI with all-cause mortality and diabetes-cardiovascular mortality, Kaplan-Meier survival curves were plotted, and Cox proportional hazards regression models were constructed. All-cause mortality and diabetes-cardiovascular mortality were significantly higher in NLR ≥ 1.516 [118 (26.5%) vs. 14 (11.8%), 52 (11.5%) vs. 5 (4.2%)], MLR ≥ 0.309 [(78 (34.1%) vs. 54 (15.7%), 36 (15.7%) vs. 21 (6.1%)], SIRI ≥ 0.756 [(114 (25.8%) vs. 18 (13.8%), 49 (11.1%) vs. 8 (6.2%)], and NLR + MLR + SIRI subgroups [(77 (34.2%) vs. 55 (15.9%), 35 (15.6%) vs. 22 (6.3%)] than NLR < 1.516, MLR < 0.309, SIRI < 0.756 subgroups, and other participants except NLR + MLR + SIRI (all *P* < 0.05) (Figs. 3 and 4). Multivariate Cox regression models 1, 2, 3, and 4 for NLR, MLR, SIRI, and NLR + MLR + SIRI of all-cause mortality and diabetes-cardiovascular mortality showed that NLR ≥ 1.516, MLR ≥ 0.309, SIRI ≥ 0.756, and NLR + MLR + SIRI were associated with all-cause mortality and diabetes-cardiovascular mortality. They may be independent predictors of all-cause mortality and diabetes-cardiovascular mortality (Table 5). The hazard ratios (HR) for NLR, MLR, SIRI, and NLR + MLR + SIRI in terms of all-cause mortality were 1.979 (1.13–3.468), 1.850 (1.279–2.676), 1.821 (1.096–3.025), and 1.871 (1.296–2.703), respectively. Similarly, the HR for NLR, MLR, SIRI, and NLR + MLR + SIRI concerning diabetes-cardiovascular mortality were 2.602 (1.028–6.591), 2.673 (1.483–4.818), 2.001 (0.898–4.459), and 2.554 (1.426–4.575), respectively. In the restricted cubic spline plots, the relationship between NLR, MLR, SIRI, and HR for all-cause mortality and diabetes-cardiovascular mortality was generally shaped like a “J” (Supplementary Fig. 2).

**Table 5:** Cox proportional hazards regression models for NLR, MLR, SIRI, NLR + MLR + SIRI of all-cause mortality and diabetes-cardiovascular mortality

Variables	Model		Model 2		Model 3		Model 4	
	HR(95%CI)	*P*	HR(95%CI)	*P*	HR(95%CI)	*P*	HR(95%CI)	*P*
All-cause mortality								
age ≥ 60 yaers	3.537(2.132–5.868)	< 0.001	3.408(2.056–5.649)	< 0.001	3.456(2.085–5.730)	< 0.001	3.417(2.061–5.663)	< 0.001
Red blood cell	1.973(1.381–2.817)	< 0.001	1.820(1.266–2.617)	0.001	2.041(1.433–2.906)	< 0.001	1.816(1.263–2.610)	0.001
Platelet count ≥ 150	1.717(1.112–2.651)	0.015	1.615(1.044–2.499)	0.031	1.852(1.200-2.858)	0.005	1.639(1.060–2.533)	0.026
RDW ≥ 14.5	1.893(1.303–2.75)	0.001	1.838(1.264–2.67)	0.001	1.800(1.235–2.623)	0.002	1.818(1.250–2.643)	0.002
NLR ≥ 1.516	1.979(1.13–3.468)	0.017						
MLR ≥ 0.309			1.850(1.279–2.676)	0.001				
SIRI ≥ 0.756					1.821(1.096–3.025)	0.021		
NLR + MLR + SIRI							1.871(1.296–2.703)	0.001
Diabetes-cardiovascular mortality								
Age ≥ 60 yaers	3.568(1.651–7.713)	0.001	3.308(1.529–7.157)	0.002	3.422(1.584–7.389)	0.002	3.333(1.541–7.208)	0.002
Red blood cell	2.641(1.163–5.996)	0.020	2.560(1.124–5.830)	0.025	2.439(1.077–5.523)	0.032	2.528(1.11–5.756)	0.027
Total cholesterol	2.41(1.362–4.264)	0.003	2.101(1.173–3.763)	0.013	2.479(1.404–4.377)	0.002	2.112(1.178–3.786)	0.012
NLR ≥ 1.516	2.602(1.028–6.591)	0.044						
MLR ≥ 0.309			2.673(1.483–4.818)	0.001				
SIRI ≥ 0.756					2.001(0.898–4.459)	0.090		
NLR + MLR + SIRI							2.554(1.426–4.575)	0.002

Considering the significant difference in age distribution between the two groups, age exhibited the highest HR value for both all-cause mortality and diabetes-cardiovascular mortality, significantly surpassing NLR, MLR, and SIRI. Age demonstrated a positive correlation with NLR, MLR, and SIRI. Consequently, we analyzed the Kaplan-Meier survival curves for all-cause mortality concerning NLR, MLR, SIRI, and NLR + MLR + SIRI in participants aged < 60 and ≥ 60 years, respectively. In both age groups (< 60 and ≥ 60 years), all-cause mortality were significantly higher in NLR ≥ 1.516, MLR ≥ 0.309, SIRI ≥ 0.756, and NLR + MLR + SIRI subgroups than NLR < 1.516, MLR < 0.309, SIRI < 0.756 subgroups, and other participants except NLR + MLR + SIRI (all *P* < 0.05) (Supplementary Figs. 3 and 4). In addition, antidiabetic drugs may affect chronic systemic inflammation, we conducted subgroup analyses on whether participants were treated with insulin, antidiabetic drugs, respectively. In participants treated with or without antidiabetc drugs, and treated with or without insulin, all-cause mortality were significantly higher in MLR ≥ 0.309, and NLR + MLR + SIRI subgroups than MLR < 0.309 and other participants (all *P* < 0.05) (Supplementary Figs. 5, 6, 7, and 8).

## Discussion

This study represents the first exploration into the relationship between inflammatory markers NLR, MLR, and SIRI and the prognosis of DR patients, to offer novel evidence for guiding the treatment of DR patients and improving their prognosis. Our analysis has revealed a significant association between elevated levels of NLR, MLR, and SIRI are associated with both all-cause mortality and diabetes-cardiovascular mortality in participants with DR, suggesting their potential utility as independent prognostic predictors for DR. Elevated levels of NLR, MLR, and SIRI were consistently linked to increased all-cause mortality and diabetes-cardiovascular mortality in participants aged < 60 and ≥ 60 years. Combining NLR, MLR, and SIRI in clinical practice may enhance predictive accuracy for these outcomes in individuals with DR.

Chronic low-grade inflammation is a pivotal driver of diabetic retinal disorders, with studies indicating its prevalence in various stages of DR in both diabetic patients and animal models [22]. Diabetic patients often experience chronic inflammation, which leads to the infiltration of inflammatory cells and subsequent tissue damage, exacerbating retinal vascular complications, such as permeability, vasodilation, and retinal thickening, in DR patients [5]. Numerous studies have demonstrated the pivotal involvement of diverse inflammatory components, including inflammatory cells (such as macrophages and lymphocytes), inflammatory cytokines (TNF-α, IL-4, and IL-10), chemokines (MCP-1, CCL2, and CCL5.), and adhesion molecules (including ICAM-1, VCAM-1, selectin, and integrin), in the indispensable processes governing the development and progression of DR [5, 23–25]. The accumulation of inflammatory cells and molecular mediators contributes to capillary dysfunction, thereby fostering the advancement of DR. NLR, MLR, and SIRI are emerging as clinically accessible, straightforward, and cost-effective alternative indicators of inflammation. In the context of diabetes, neutrophils primarily release inflammatory molecules that affect blood vessel integrity, and monocytes discharge various hormones and cytokines influencing insulin secretion and function. In contrast, lymphocytes primarily function as regulators of inflammatory activity [26, 27].

NLR serves as a reliable indicator of inflammation across various metabolic diseases, with elevated levels observed in numerous inflammatory diseases, including type 2 diabetes, irritable bowel disease, and heart disease [28, 29]. It is used to predict the prognosis of inflammatory diseases such as cardiovascular disease, gestational diabetes mellitus, and chronic obstructive pulmonary disease [16, 30]. A meta-analysis has substantiated the significant association between increased NLR levels and poor glycemic control in type 2 diabetes mellitus (T2DM) patients, indicating the inflammatory burden of diabetes. NLR has been observed to increase with the deterioration of HbA1c [31]. Elevated NLR levels, attributed to lymphocytopenia and neutrophilia, are linked to microvascular and macrovascular complications as well as metabolic damage in diabetes [31]. Previous studies have suggested the association of NLR with brachial-ankle pulse wave velocity and an increased risk of DR, suggesting its applicability as a marker of DR [32, 33]. However, there is currently no available research on the prognosis of NLR and DR. Our study demonstrated that NLR was associated with all-cause mortality and diabetes-cardiovascular mortality in participants with DR, aligning with the trend of NLR serving as a prognostic factor in other diseases.

MLR, a novel inflammatory index, has been shown to effectively reflects the diverse clinical conditions of DR patients [34]. Chemokines play a crucial role in recruiting and activating monocytes and leukocytes in DR, and their production contributes to the elevation of MLR [35, 36]. Studies have indicated that MLR is associated with an increased risk of proliferative DR and serves as a reliable indicator of DR [31, 36]. MLR is widely recognized as an effective prognostic indicator for various diseases, including patients with T2DM and chronic kidney disease [37], as well as those undergoing peritoneal dialysis [19]. In a study by Huang et al. involving 307 diabetic patients, MLR was suggested to be related to the occurrence and severity of DR. An analysis of 95 DR patients found that MLR may be a useful predictor of DR progression [20]. These findings align with our results, with a larger sample size, and further verify that MLR could be a predictor of DR.

SIRI is a novel inflammatory marker that reflects the state of the systemic inflammatory response, initially proven to be a prognostic indicator of adverse outcomes in cancer patients [38, 39]. Han et al. [40] found that SIRI was a predictive biomarker for major adverse cardiovascular events in acute coronary syndrome patients after percutaneous coronary intervention, potentially surpassing NLR and MLR [41]. Previous studies have shown a correlation between SIRI and cardiovascular disease and its prognosis. Elevated SIRI levels were positively associated with myocardial infarction, coronary artery disease severity, and an increased risk of stroke and all-cause mortality, providing new insights into the assessment of mortality risk [39, 42, 43]. However, there is limited evidence linking SIRI to diabetes. A recent study suggested that SIRI was independently associated with peripheral artery disease and its severity in diabetic patients [44]. In this study, we found for the first time that SIRI is related to the prognosis of patients with DR and may serve as a prognostic predictor.

Furthermore, we evaluated the individual and collective predictive efficacy of NLR, MLR, and SIRI to evaluate their individual and collective predictive efficacy in clinically predicting all-cause mortality. The AUC and decision curve analysis indicated that MLR and NLR + MLR + SIRI slightly outperformed NLR and SIRI in predicting all-cause mortality. In clinical practice, the combination of NLR, MLR, and SIRI may be utilized to predict both all-cause mortality and diabetes-cardiovascular mortality in participants with DR. Therefore, these markers, NLR, MLR, and SIRI, may be employed in clinical settings to anticipate the clinical outcomes of patients with DR, providing valuable guidelines for medical practitioners.

This study was based on a comprehensive population-based survey dataset from NHANES, with a sampling design that ensures the representativeness of our results for the entire population. Nevertheless, our study has certain limitations that warrant acknowledgment. First, due to the observational study design, only correlation, not causation, could be obtained. Second, this study lacked detailed information on the severity of diabetes, potentially introducing bias into our conclusions. Third, We have not considered renal function status, another manifestation of diabetic microangiopathy, for the outcomes. Finally, the possibility of residual and unknown confounding cannot be entirely excluded, such as the inflammatory biomarkers (NLR, MLR and SIRI) are not specific for DR and may also be elevated in other inflammatory diseases.

## Conclusion

NLR, MLR, and SIRI may serve as three independent prognostic predictors for all-cause mortality and diabetes-cardiovascular mortality in individuals with DR. In practical clinical practice, combining NLR, MLR, and SIRI could improve the predictive accuracy of all-cause mortality and diabetes-cardiovascular mortality in participants with DR, These results may facilitate the development of early intervention strategies, thereby enhancing clinical outcomes and improving patient prognosis.

### Electronic supplementary material

Below is the link to the electronic supplementary material.

Supplementary Material 1

## Data Availability

No datasets were generated or analysed during the current study.
