# Supplementary material for: The association of inflammatory biomarkers with clinical outcomes in diabetic retinopathy participants: data from NHANES 2009–2018
Source: Diabetol Metab Syndr. 2024 Jul 29;16:181. doi: 10.1186/s13098-024-01419-4 (PMC11285410; doi:10.1186/s13098-024-01419-4)
Supplement: Supplementary file 1 — Supplementary Material 1 [file 13098_2024_1419_MOESM1_ESM.pdf]

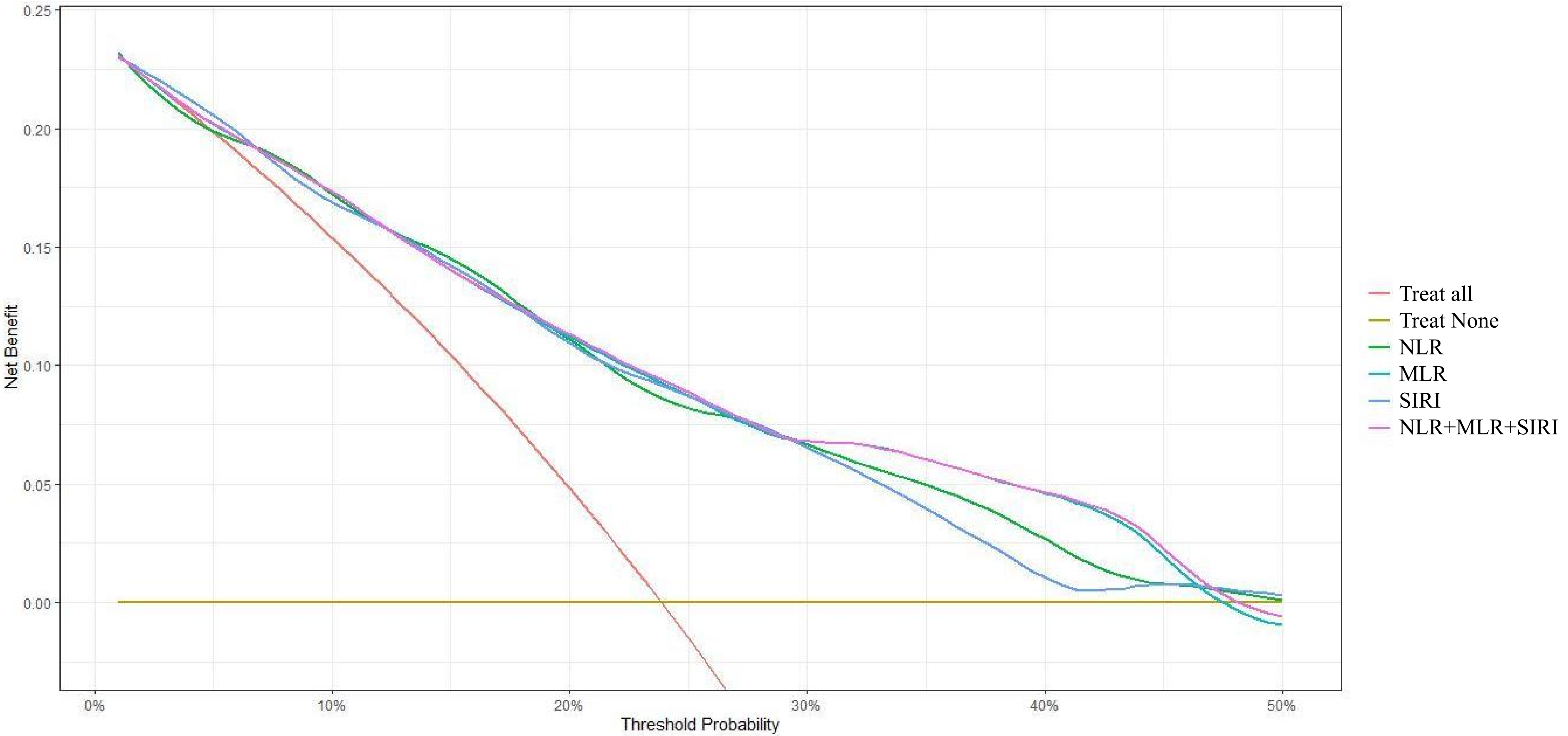

Supplementary Figure 1. The Decision Curve Analysis curves of NLR, MLR, SIRI, and NLR + MLR + SIRI for all-cause mortality.

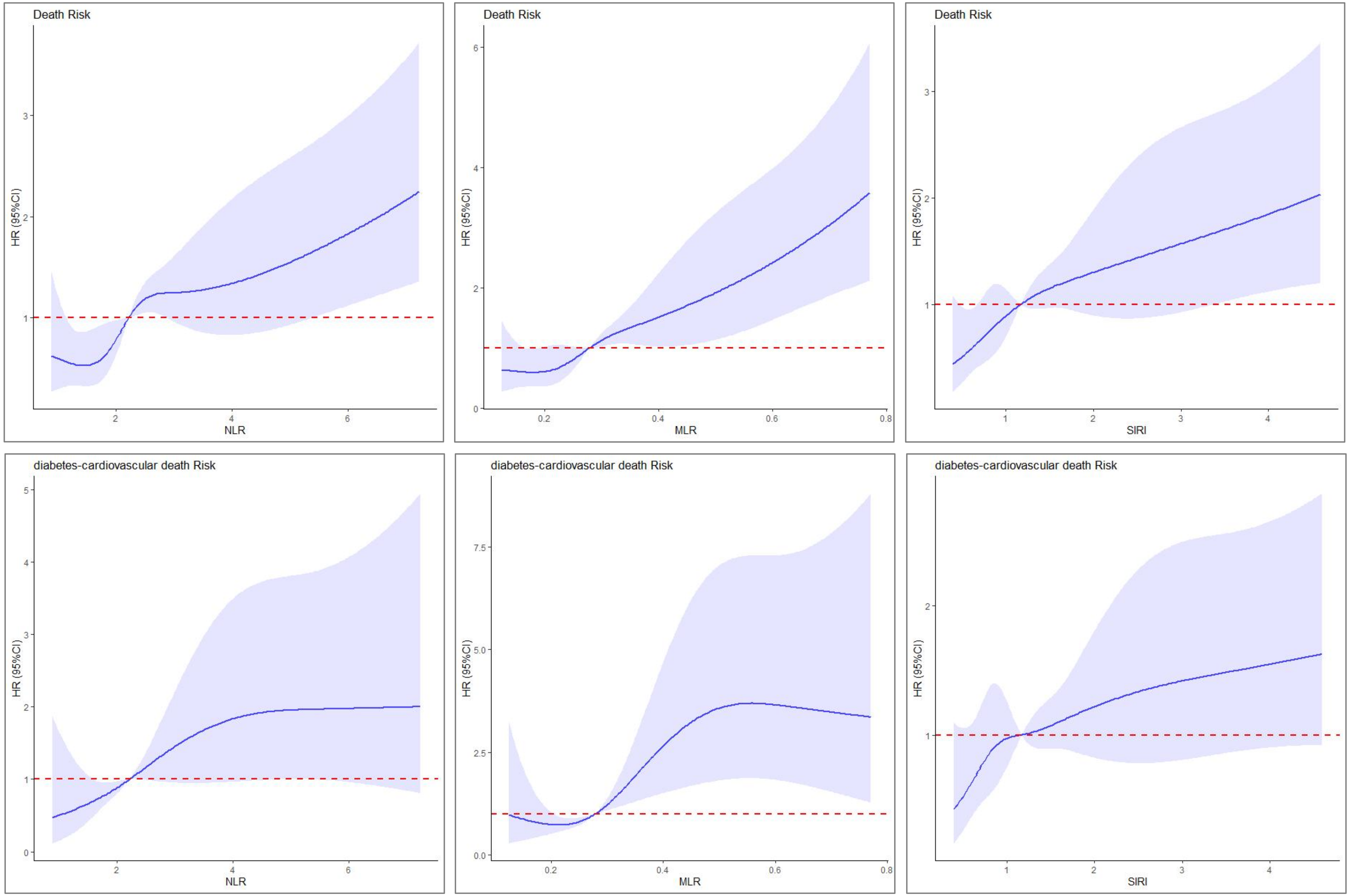

Supplementary Figure 2. The restricted cubic spline plots of NLR, MLR, and SIRI for all-cause mortality and diabetes-cardiovascular mortality.

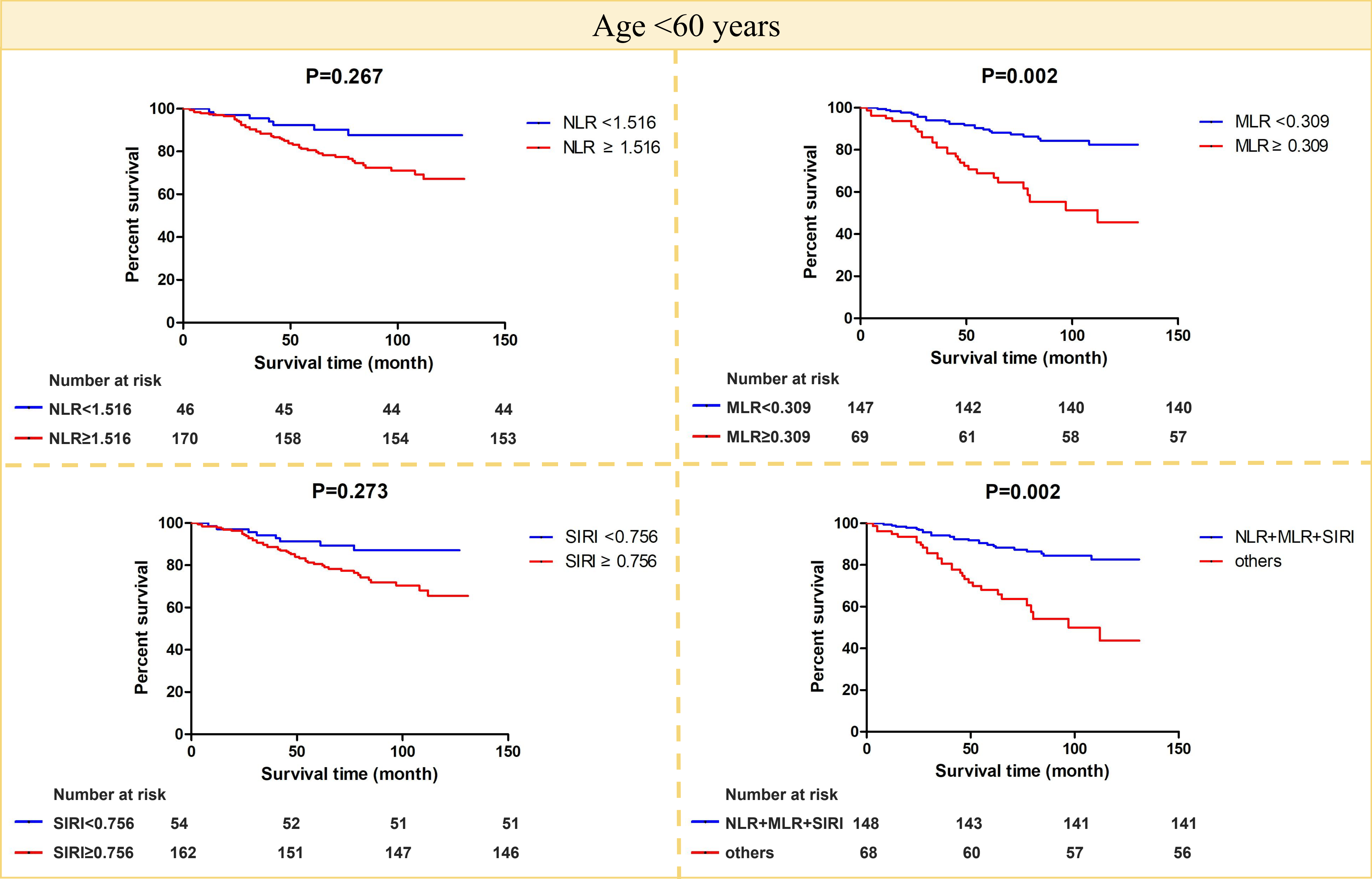

Supplementary Figure 3. The Kaplan-Meier curves for all-cause mortality for NLR, MLR, SIRI and NLR + MLR + SIRI in age <60 years participants, respectively.

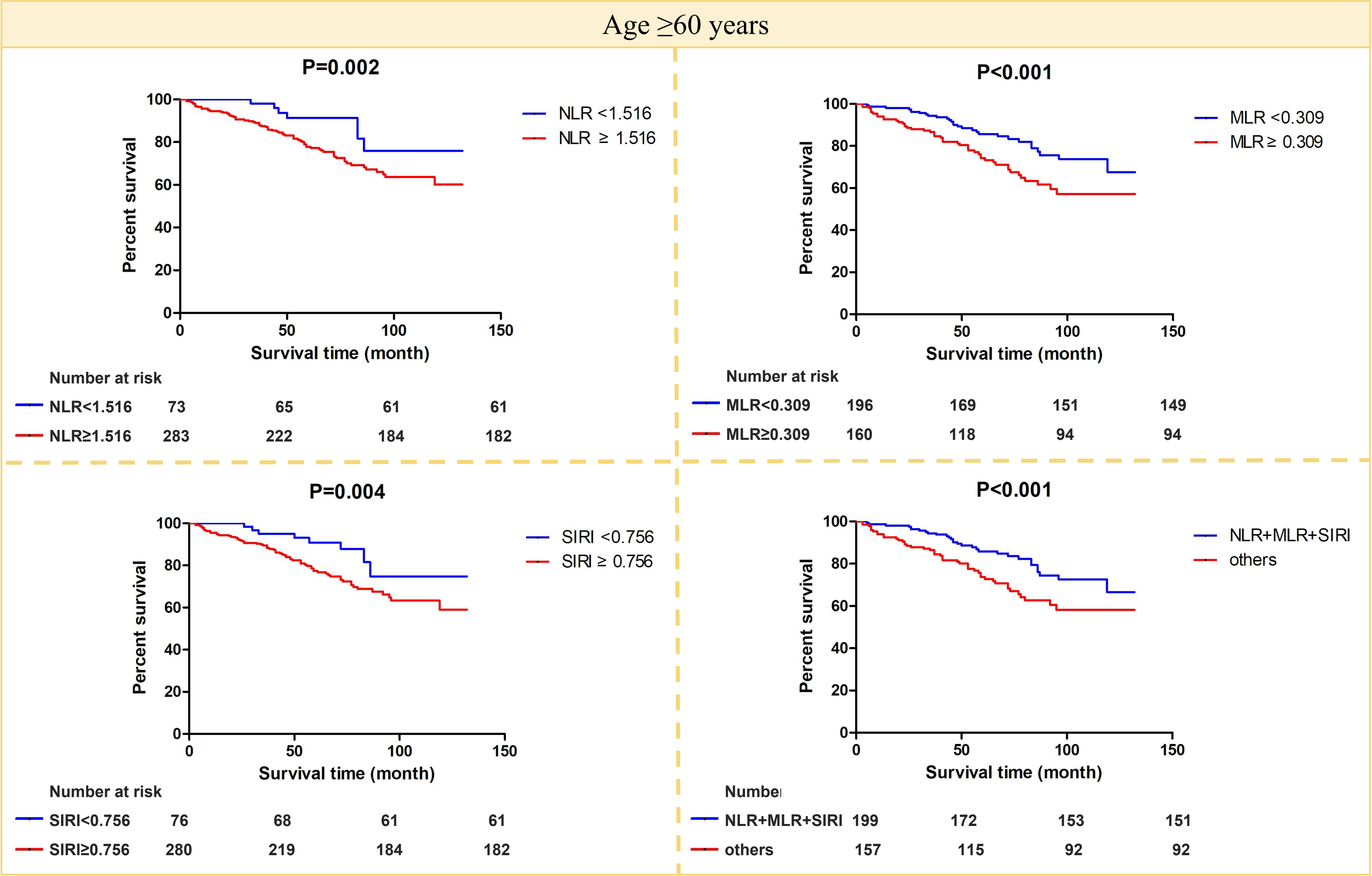

Supplementary Figure 4. The Kaplan-Meier curves for all-cause mortality for NLR, MLR, SIRI and NLR + MLR + SIRI in age ≥60 years participants, respectively.

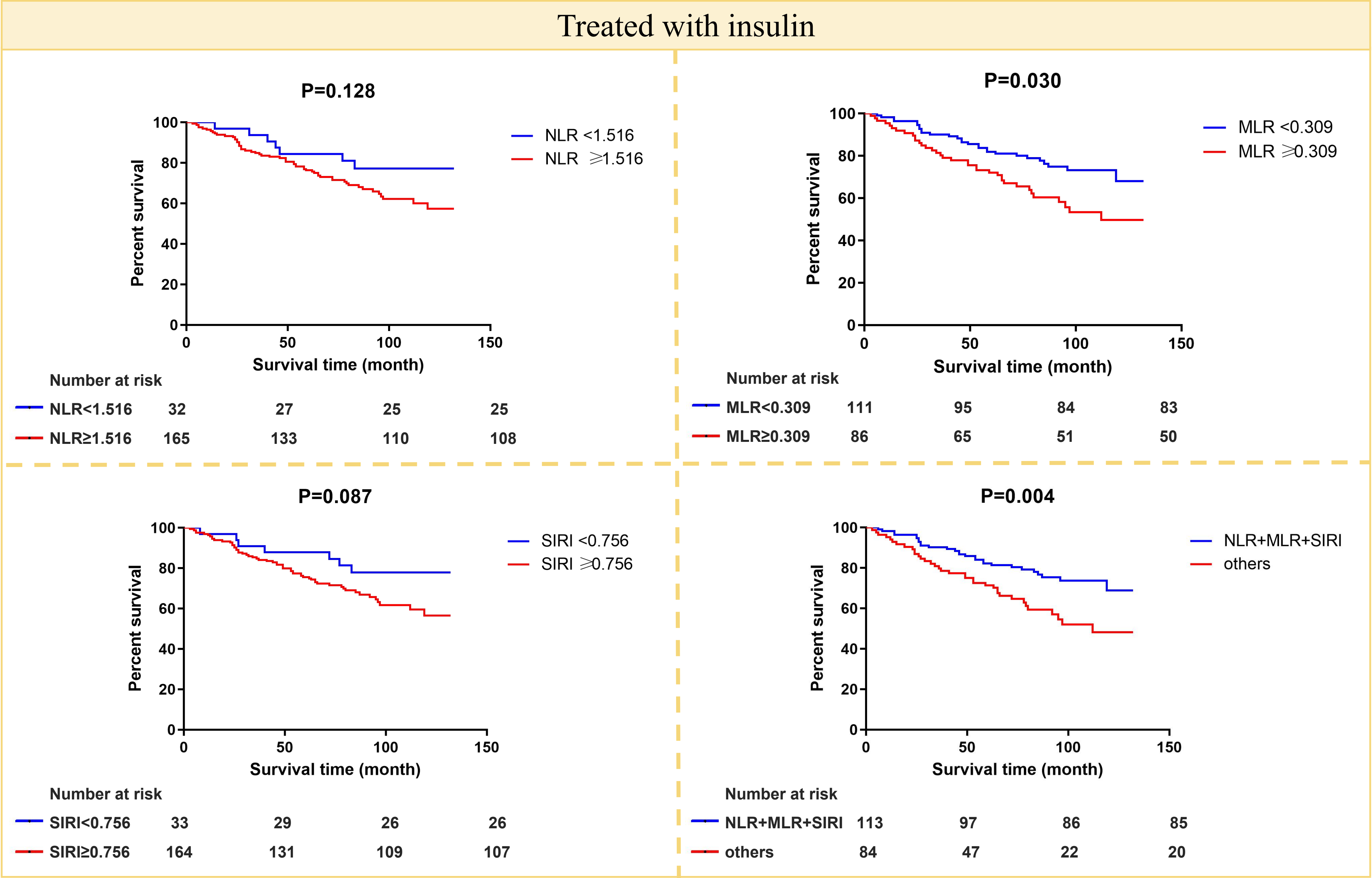

Supplementary Figure 5. The Kaplan-Meier curves for all-cause mortality for NLR, MLR, SIRI and NLR + MLR + SIRI in participants treated with insulin, respectively.

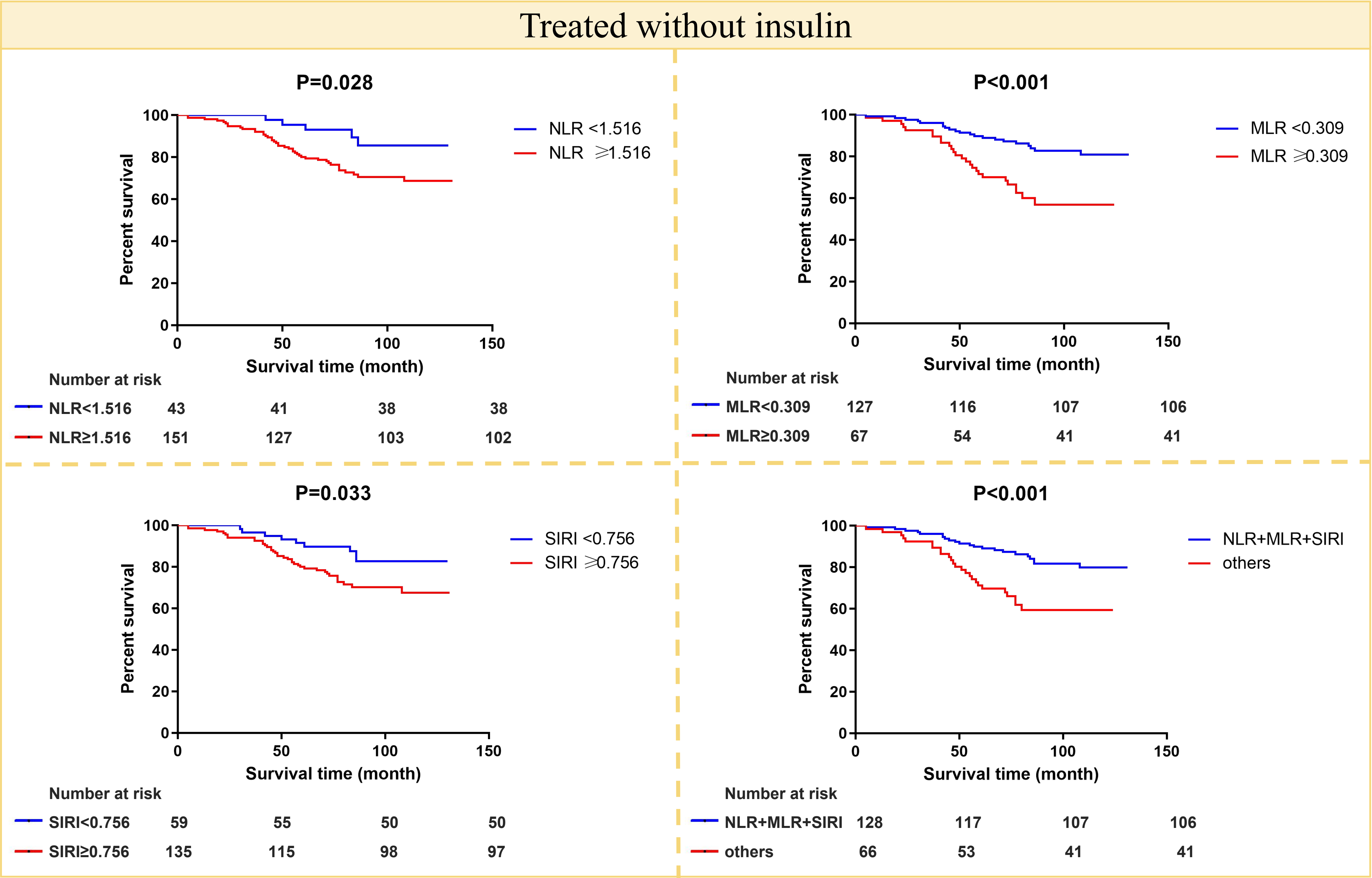

Supplementary Figure 6. The Kaplan-Meier curves for all-cause mortality for NLR, MLR, SIRI and NLR + MLR + SIRI in participants treated without insulin, respectively.

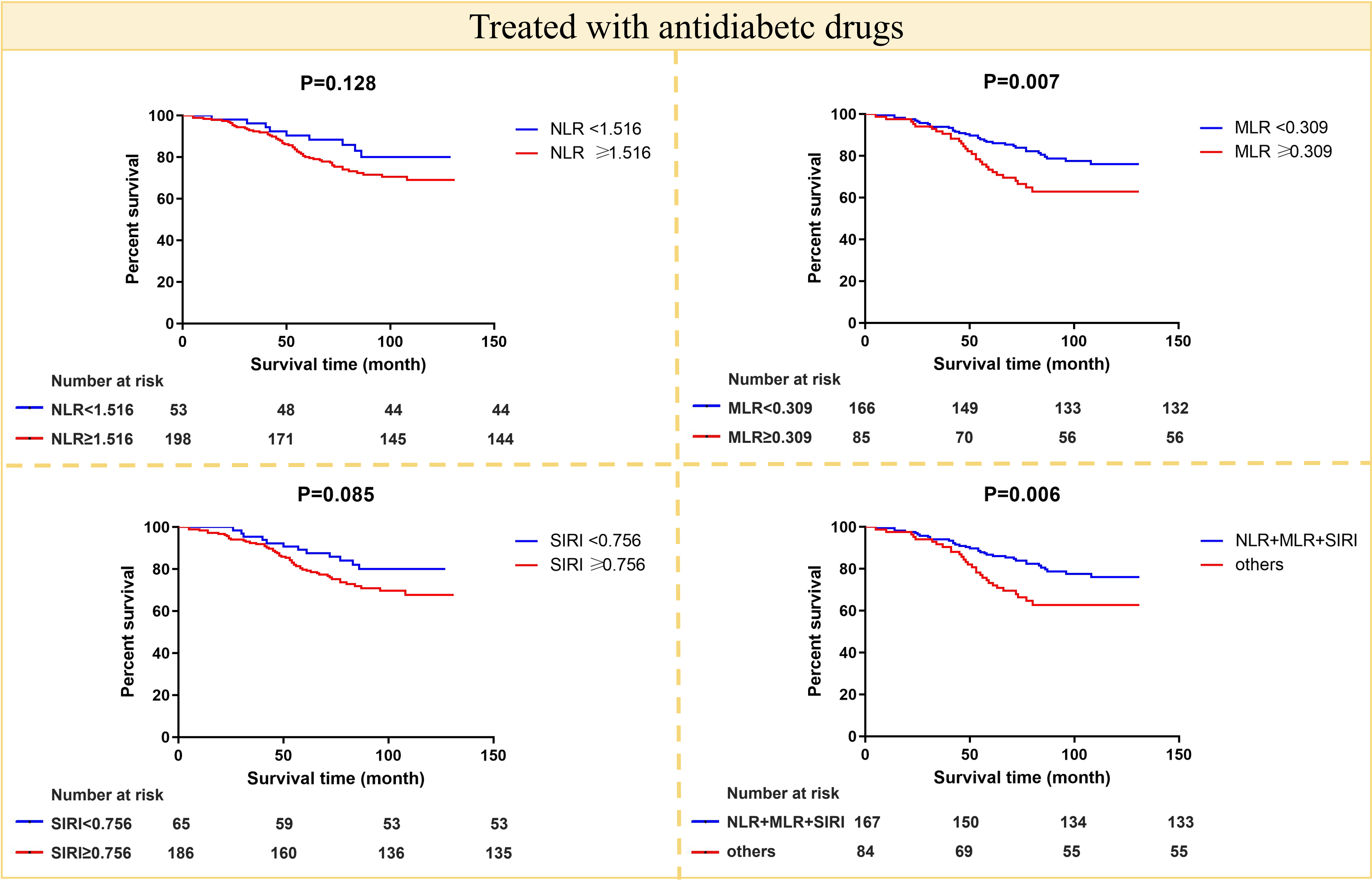

Supplementary Figure 7. The Kaplan-Meier curves for all-cause mortality for NLR, MLR, SIRI and NLR + MLR + SIRI in participants treated with antidiabetc drugs, respectively.

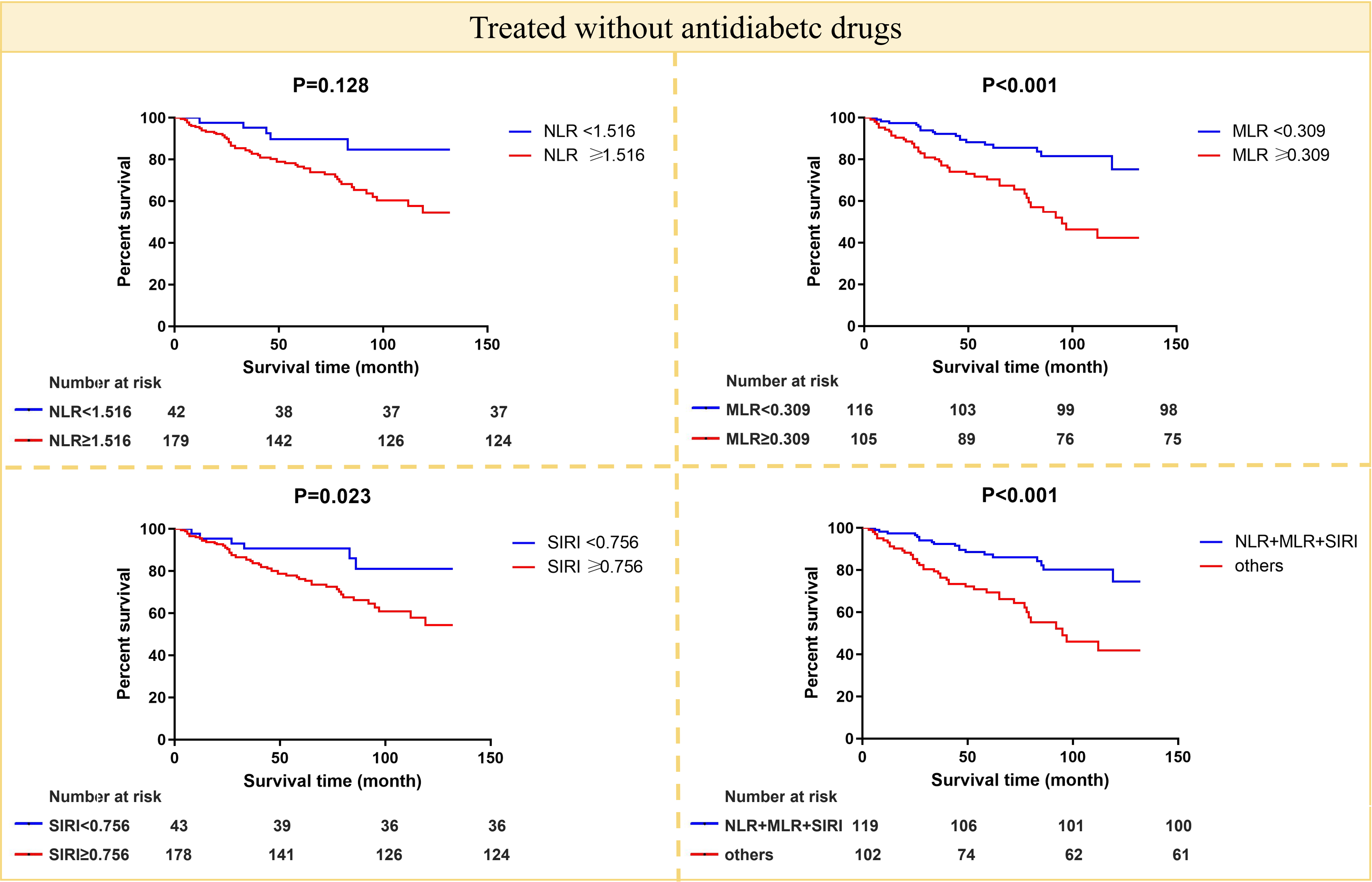

Supplementary Figure 8. The Kaplan-Meier curves for all-cause mortality for NLR, MLR, SIRI and NLR + MLR + SIRI in participants treated without antidiabetc drugs, respectively.

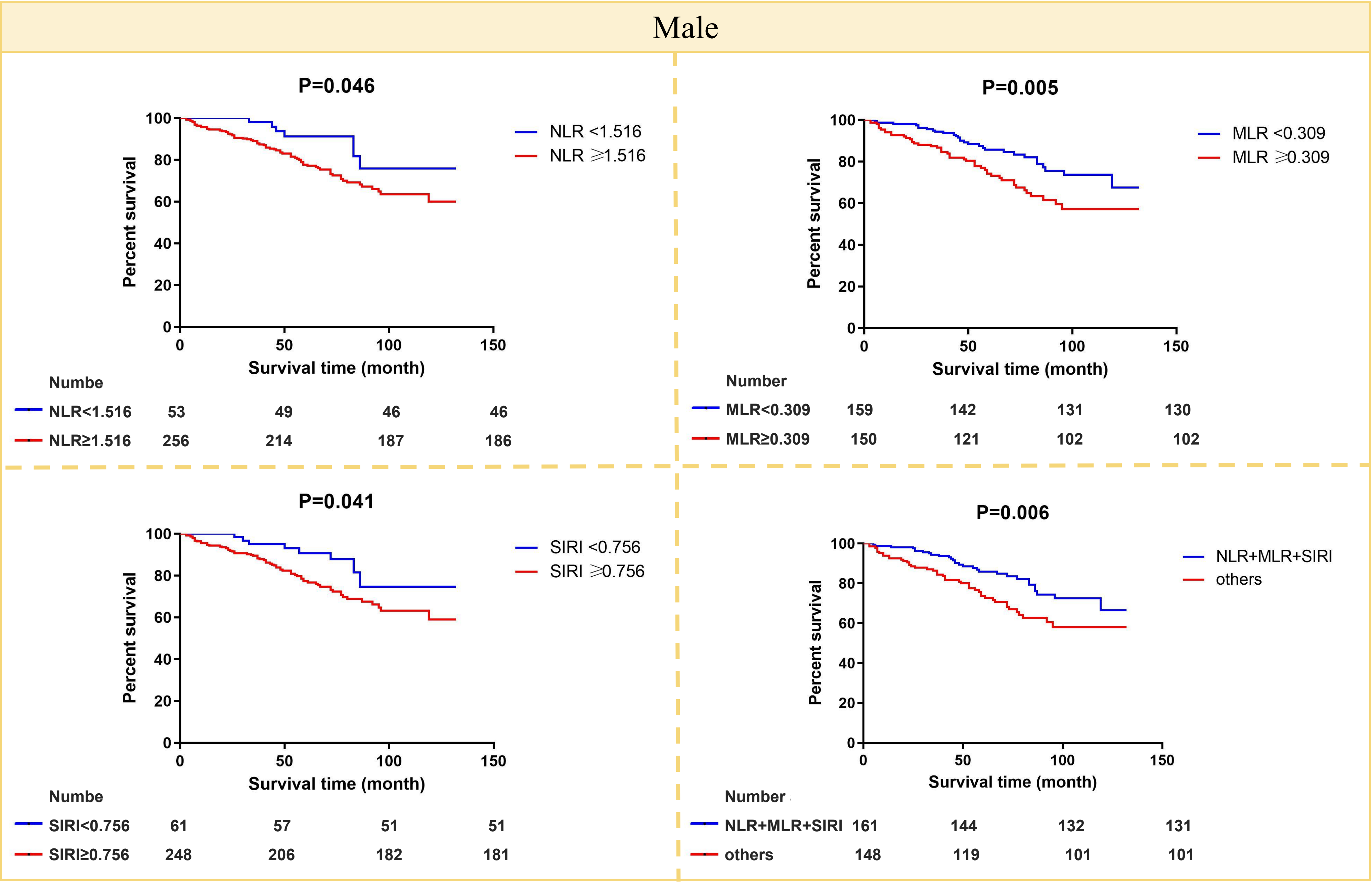

Supplementary Figure 9. The Kaplan-Meier curves for all-cause mortality for NLR, MLR, SIRI and NLR + MLR + SIRI in male participants, respectively.

Female

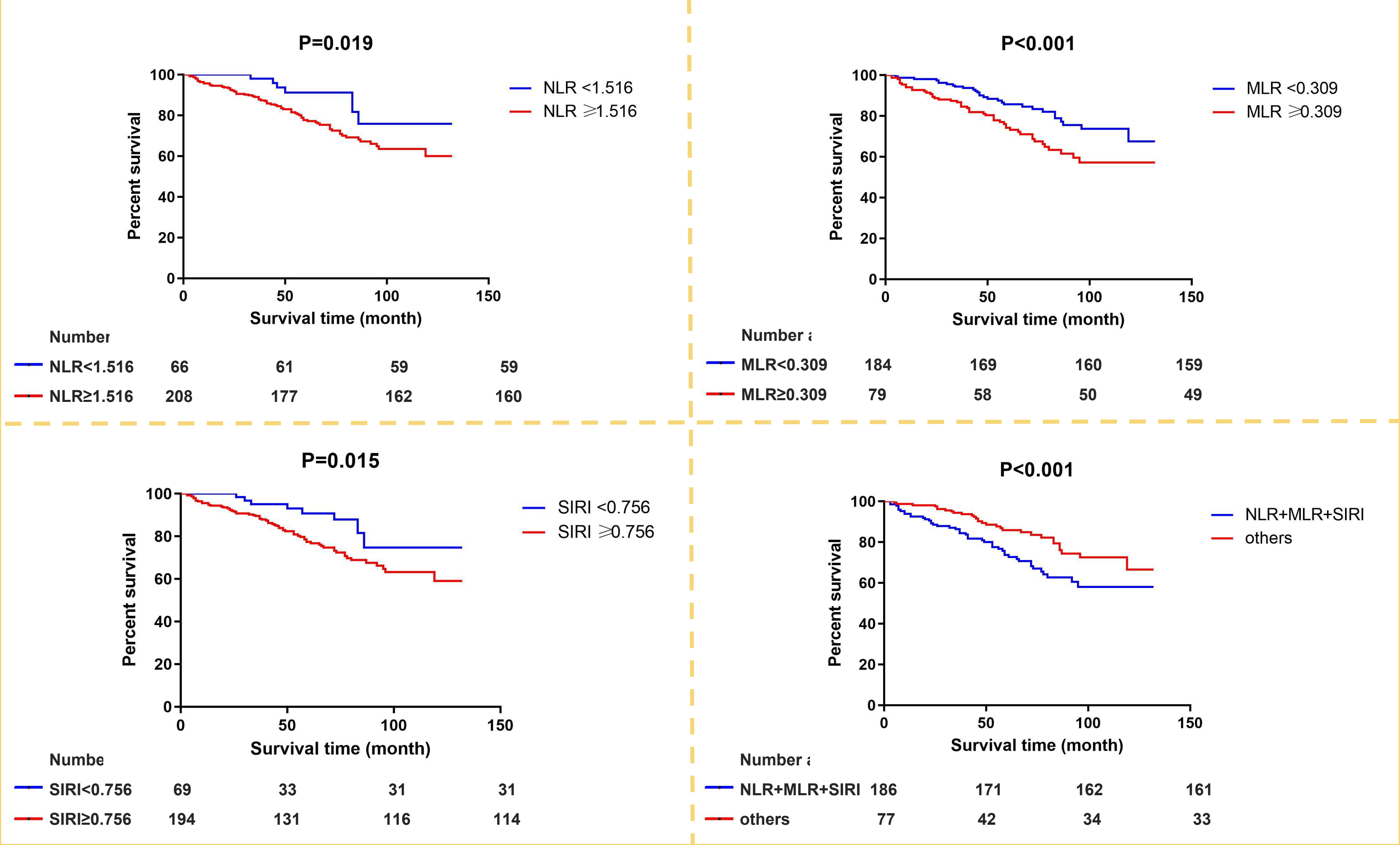

Supplementary Figure 11. The Kaplan-Meier curves for all-cause mortality for NLR, MLR, SIRI and NLR + MLR + SIRI in female participants, respectively.
